# Redox Potential as a Means to Control the Treatment of Slurry to Lower H*_2_*S Emissions

**DOI:** 10.3390/s120505349

**Published:** 2012-04-26

**Authors:** Maibritt Hjorth, Christina Ø Pedersen, Anders Feilberg

**Affiliations:** Faculty of Science and Technology, Department of Engineering, Aarhus University,Research Centre Foulum, Blichers Allé 20, 8830 Tjele, Denmark;E-Mails: Christina.Osterballe@agrsci.dk (C.P.); Anders.Feilberg@agrsci.dk (A.F.)

**Keywords:** animal manure, hydrogen sulfide, nitrogen, odor, ORP, oxidation curve, oxygen, ozone

## Abstract

Slurry can be oxidized to eliminate undesirable emissions, including malodorous hydrogen sulfide (H*_2_*S). However, it is difficult to assess the optimal amount of oxidizing agent required. In this study, one cow and one pig manure, each in three particle size ranges were oxidized with 0–350 mg ozone/L manure. Redox and H*_2_*S concentration were measured continuously. During ozonation the manures gave equivalent redox potential curves. A relatively rapid rise in redox potential was observed within a range of −275 mV to −10 mV, with all manures changing as a minimum from −200 mV to −80 mV. The gaseous H*_2_*S emissions were decreased by 99.5% during the redox increase (−200 mV to −80 mV). This is attributed to H*_2_*S oxidation by ozone and oxygen, and is not due to H*_2_*S deprotonation or gas flushing. By identifying the initiation of the final redox level following the rise, the amount of ozone required to remove H*_2_*S from the manure samples was estimated to be in the range of 6–24 mg O*_3_*/L manure, depending on the type of manure. Hence, continuous monitoring of redox potential (termination of the redox rise) during the oxidation treatment is a simple method of achieving cost-effective minimization of H*_2_*S emissions from slurry.

## 1. Introduction

Malodorous emissions from liquid wastes such as animal manure and wastewater are a nuisance to the nearby communities. Oxidation can be carried out to lower the odor emissions [1,2]. Hydrogen sulfide (H*_2_*S) is a major contributor to odor from slurry, e.g., during agitation and field application [3,4] and pig production facilities [5–7]. Additionally, H*_2_*S poses a health risk to animals and people. H*_2_*S can be converted to less volatile and odorous sulfur compounds by oxidation, and the emissions of H*_2_*S are therefore minimized [8–10].

To remove odor from animal manure, ozone has previously been added as an oxidizing agent [5,11,12]. Due to the high reactivity of ozone [13], it can be expected to be very effective for the H*_2_*S oxidation compared to other oxidizing agents. Ozone is also added for other reasons including antibacterial treatment and removal of other odorous compounds [11], hence ozone treatment is an appealing process for several reasons. A broad range of ozone doses has previously been applied; from 250 to 3,000 mg O*_3_*/L manure [1,8,11,12]. However, overdosing must be avoided for economic reasons and additionally for safety reasons. Little information on the optimal dose of oxidizing agent for obtaining the most cost-effective treatment is available, and it is not easy to assess. Hence, to enable more cost-effective treatments and concurrently guaranteeing abatement of malodourous H*_2_*S emissions, a sensor for process control is required.

Analytical methods used to observe the required ozone dose have included olfactometry, quantification of the emitted odorous compounds, quantification of precursors of odorous gases in the slurry, the predominant microbial population, and redox potential [1,9,14,15]. Of these, redox potential is the simplest measurement, performed with an electrode.

In effluents such as wastewater and animal manure [10,16], many components can be oxidized, including H*_2_*S, NH*_4_*^+^, carboxylic acids, phenols, and larger organic components. The different chemical components have different standard electrode potentials, different reaction rates and different reactions with the oxidizing agents. Hence, the components may be oxidized in a specific order, and this may result in an oxidation curve (amount of oxidizing agent *vs.* redox potential) consisting of different levels. The concentrations of the chemical components affect the amount of oxidizing agent required to obtain a specific redox potential. Previous studies have observed a cessation of H*_2_*S emissions: (i) between −100 mV and 0 mV in manure, (ii) between −100 mV and −50 mV in sediment, and (iii) at −208 mV in wastewater [9,17,18]. Thus, if the cessation of H*_2_*S emissions have a specific relationship with a quantifiable level of the oxidation curve, a redox electrode could be a suitable sensor for process control.

The aim of this study was therefore to explore: (i) the general oxidation curve (amount of oxidizing agent *vs.* redox potential), (ii) the amount of H*_2_*S emissions in relation to the oxidation level, (iii) the chemical reactions and (iv) the possibility of using redox measurement as a simple method for assessing the required amount of oxidizing agent. Six different batches of animal manure were oxidized with ozone and oxygen. The redox potential and the amount of H*_2_*S emissions were measured continuously.

## 2. Experimental Section

### 2.1. Manure Samples

One batch of sow manure and one batch of dairy cattle manure were collected from two commercial farms. To obtain three manure samples per animal type with different amounts of potentially ozone reactive compounds, two separation techniques were applied to both manures.

One part of each manure was separated on-site with a commercial farm-scale screw press (SB Engineering, Aalestrup, Denmark) using a 250 µm filter.

One part of the sow manure was separated in a commercial farm-scale unit (AL-2 Teknik, Hovborg, Denmark) using polymer flocculation and filtration. One part of the cattle manure was separated in 0.6-L batches in the laboratory using polymer flocculation and filtration. Cationic, high molecular weight, linear polyacrylamide was used in both treatments. The optimal polymer charge density and dosage were determined based on floc size, dewaterability, volume separation, liquid turbidity, and solid dry matter content [19]. For the sow and cattle manures, 0.45 mL of 50% Superfloc c-2260 emulsion (40 mol% charge density) and 1.7 mL of 50% Superfloc c-2240 (20 mol% charge density) emulsion (Cytec, NJ, USA) were used per liter of manure, respectively. The sow manure was sieved on a 250 µm roller belt filter, and pressure was applied using a drum roller. The cattle manure was sieved through a 200µm filter, and no pressure was applied.

Six samples were therefore obtained: one raw sow manure (s1), one screw-pressed liquid from the sow manure (s2), one flocculated liquid from the sow manure (s3), one raw cattle manure (c1), one screw-pressed liquid from the cattle manure (c2), and one flocculated liquid from the cattle manure (c3).

### 2.2. Oxidation Treatment

Vessels (5 L, Ø 170 mm) were filled with 2.5 L batches of manure, and the manure was continuously stirred. Two different stirring methods were used. Tests proved that they resulted in equal effects on redox potential and H*_2_*S emissions. The sow manure was stirred with an overhead stirrer (RZR 2041; Heidolph, Schwabach, Germany) and an impeller (BR 13; Heidolph) at 180 rpm. The cattle manure was stirred manually by spinning the gas inlet diffuser at ∼85 rpm.

An ozone-enriched oxygen stream was generated by passing pure oxygen through an electrical discharge ozone generator (Ozonia LAB2B; Degrémont Technologies–Triogen, East Kilbride, Scotland). The ozone generator was fed oxygen from a gas cylinder at a flow of 2 L/min at a pressure of 1.5 bar. The generator was set to its maximum output level, resulting in a gas containing 1.5–2.2% O*_3_* and 97.8–98.5% O*_2_*. The ozone concentration was measured before each experiment with an ozone analyzer (UV-100; Eco Sensors, Fresno, CA, USA), using pre-dilution in order to be within the analytical range. The ozone-enriched oxygen stream was led through Teflon tubes, and injected at the bottom of the manure-filled vessel through a stainless steel solvent filter (A-230A; Upchurch Scientific, Oak Harbor, WA, USA, pore size 20 µm) used as a diffuser for dispersion of the added gas. The O*_2_*/O*_3_* mixture was added continuously at a constant rate, for the various experiments at 0.075–0.18 L/min. Depending on outlet ozone concentration measured before each experiment, the addition rate was 2.5–5.5 mg O*_3_*/min. The maximum ozone addition was 350 mg O*_3_*/L manure and 17,000 mg O*_2_*/L manure. To apply different ozone doses, the treatment time of the 2.5-L sample was varied between 10 and 160 min. The ozone was fully dissolved and consumed in the manure for all treatments. This was ensured through continuous measurement of the ozone level above the surface of the manure.

Additionally, two control treatments were performed on another pig manure by adding N*_2_* and O*_2_*. For the N*_2_* treatment, pure nitrogen was added at 0.06 L/min for 80 min, and for the O*_2_* treatment pure oxygen was added at 0.07 L/min.

### 2.3. Chemical Analyses

The analyses performed on the non-ozonated manure samples were: pH, particle sizes, total dry matter, total chemical oxygen demand (COD), volatile fatty acids, total NH*_3_*/NH*_4_*^+^ concentration and total H*_2_*S/HS^−^/S^2-^ concentration. Particle size range was analyzed using laser diffraction (Master Sizer 2000; Malvern Instruments Ltd, Worcestershire, UK). pH was measured using a pH electrode (InPro450IVP/PT100 SG; Mettler-Toledo, Zurich, Switzerland). Total dry matter was determined gravimetrically as the weight loss upon heating the sample to 105 °C (APHA, 1992). The total COD concentration was determined by performing destruction, color reaction, and spectrometric quantification using the Spectroquant Kit 114555 (Merck KGaA, Darmstadt, Germany). The total NH*_3_*/NH*_4_*^+^ concentration were measured by performing color reaction and spectrometric quantification using the Spectroquant Kit 100683 (Merck KGaA). The volatile fatty acids, including butanoic acid, were measured on a gas chromatograph [20] The total H*_2_*S/HS^−^/S^2−^ concentration was measured by precipitating with zinc, capture of the H*_2_*S gas, color reaction and spectrometric quantification using the method described by Eriksen *et al.* [21].

The continuous analyses performed during treatment were: manure pH, manure redox, gaseous H*_2_*S concentration, and gaseous ozone concentration. The pH and redox electrodes were submerged 5 cm below the surface of the stirred manure. To measure the pH and redox of the manure continuously, the pH electrode and an oxidation reduction potential (ORP) electrode (Pt4805-DXK-S8/120; Mettler-Toledo/Thornton, Switzerland) was used. The inlet tubes for the gaseous ozone and H*_2_*S concentration analyses were placed 10cm above the manure surface and 10 cm below the vessel top. Ozone and H*_2_*S emissions were measured continuously using the ozone analyzer and a gold film detector (Jerome 631x; Arizone Instruments LLC, Chandler, AZ, USA), respectively. The detection limits of the apparatus used for the H*_2_*S emissions analyses were >0.001 ppm and <45 ppm. When lower and higher levels were detected, the values of 0.001 ppm and 45 ppm, respectively, were logged.

### 2.4. Data Analysis

In total, 29 experiments with additions of ozone enriched oxygen were performed. The number of treatment replicates for the individual manure samples s1, s2, s3, c1, c2, and c3 were 5, 4, 4, 6, 6, and 4, respectively. Measurement of H*_2_*S was performed on 25 treatments. The number of treatment replicates for the individual manure samples s1, s2, s3, c1, c2, and c3 were 4, 4, 4, 5, 5, and 3, respectively.

Mean curves of “ozone amount *versus* redox potential” and “redox potential *versus* gaseous H*_2_*S concentration” were plotted using a moving average trendline with the number of points being equal to the number of replicates.

**Figure 1.**
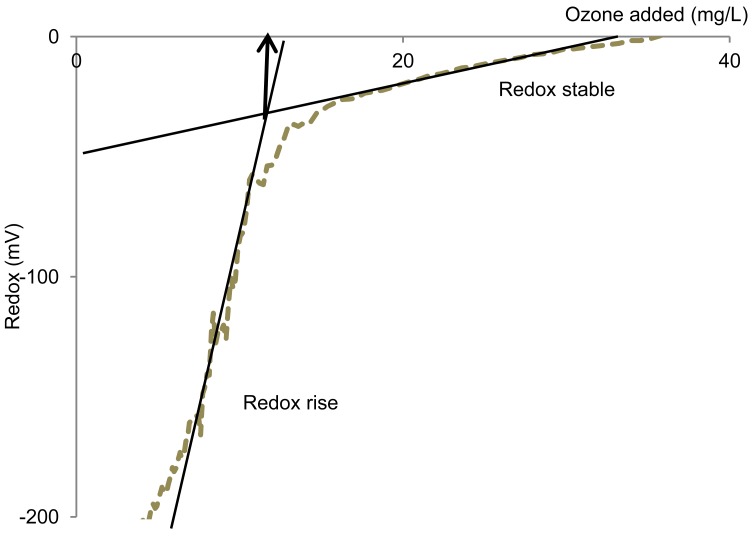
Estimation of ozone amount required for minimizing the H*_2_*S emissions, *i.e.*, the intercept of the linear regression for two of the observed redox phases: the redox rise and following relatively stable redox level. Example: manure c2.

The amount of ozone required to minimize H*_2_*S emissions was calculated as the intercept of the linear regression for two of the observed redox phases: the redox rise and the final stable redox phase (Figure 1). The ozone amounts were compared by applying Student's *t*-test (2-sided, α = 0.05).

## 3. Results and Discussion

### 3.1. Oxidation Levels

**Figure 2.**
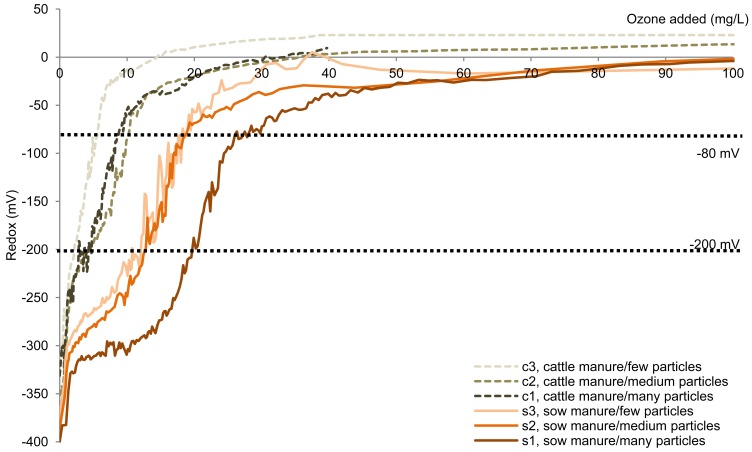
Oxidation curves upon addition of ozone enriched oxygen of the six manure samples (*n* = 4–6).

Six animal slurry batches in three particle size ranges (Table 1) were oxidized using ozone enriched oxygen in order to remove malodorous H*_2_*S emissions. The two full slurries (s1 and c1) were different in the composition. Separation caused the particle ranges to change, as did also the content of particulate compounds. The content of soluble compounds such as H*_2_*S/HS^−^ were also affected, as these can be adsorbed to the negative particles, acting as particle counterions, adsorbed to their cationic polymer or dissolved in the water retained in the solid fraction. Hence, the batches contained different amounts of potentially ozone reactive compounds. Despite the differences between the batches, the oxidation profiles exhibited similar rises in redox potential (Figure 2).

A relatively rapid rise in redox potential was observed approximately from −275 mV to −10 mV (Figure 2). Although the specific redox potential profiles varied, all exhibited as a minimum a rapid rise from −200 mV to −80 mV. Hence, similar reactions occur in the manure samples. The amount of ozone enriched oxygen added during this rise differed between the six manure batches. The redox potential increase was followed by a phase with relatively constant redox potential, which was observed to continue until the experimental maximum additions, *i.e.*, up to 350 mg O*_3_*/L manure.

### 3.2. Gaseous H2S Decrease

**Figure 3.**
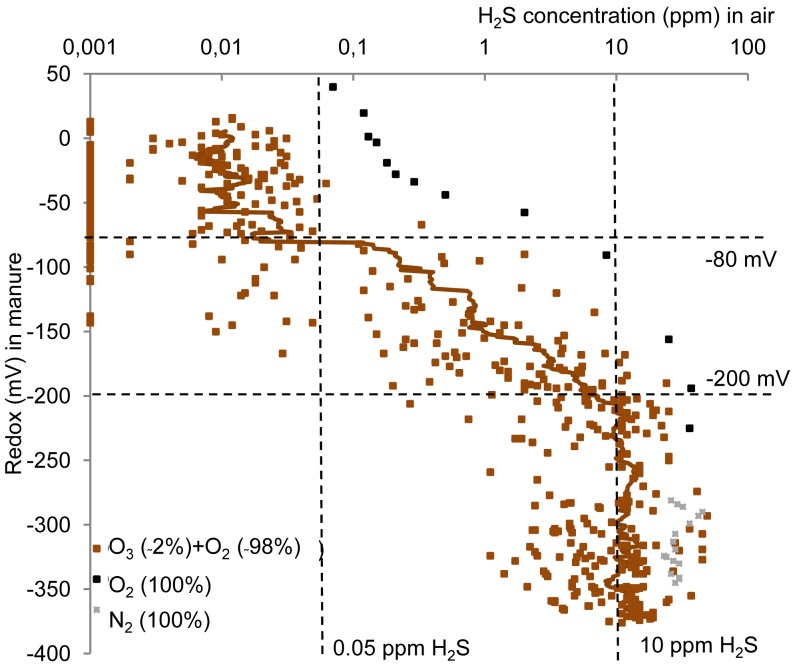
Decrease in gaseous hydrogen sulfide concentrations as a function of redox potential for N*_2_* (n = 1), O*_2_* (n = 1) and all O*_3_* treatments (n = 25).

The gaseous concentration of H*_2_*S above the manure changed as a function of addition of the ozone enriched oxygen along with the change in redox potential (Figure 3). Reductions in concentration were also observed upon addition of pure oxygen, whereas N*_2_* addition did not cause reductions in the H*_2_*S emissions.

The measured H*_2_*S emissions upon the treatment with ozone enriched oxygen remained constant at approximately 10 ppm from −400 mV until −200 mV; however the measurements were limited by the upper detection limit of the analysis equipment of 45 ppm. The H*_2_*S concentration in general decreased from this level to <0.05 ppm at around −80 mV (Figure 3). This is equivalent to a decrease in H*_2_*S emissions of more than 99%.

Previous studies have indicated that the H*_2_*S emissions were depleted between −208 mV and 0 mV [9,17,18]. This is largely in agreement with the present findings of reductions in H*_2_*S emissions around −80 mV upon ozone addition.

The decrease in H*_2_*S emissions upon addition of ozone enriched oxygen (Figure 3) occurred simultaneously with the observed redox potential rise (Figure 2), which for all samples was observed from −200 mV to −80 mV. The ozone amount that had been added when this change took place differed between the six manure samples. For all samples, however, the amount of ozone needed for reducing gaseous H*_2_*S concentration (corresponding to a rise in redox potential from −200 mV to −80 mV) was low compared with the total amount of ozone added when the minimum H*_2_*S emissions were obtained (25–65%).

### 3.3. H_2_S Reactions

The observed decrease in the gaseous concentration of H*_2_*S above the manure is ascribed to removal of H*_2_*S in the manure. This could be through oxidation by ozone, but it may also occur by oxidation by molecular oxygen, microbial oxidation, acid-base reaction and volatilization during gas addition.

**Figure 4.**
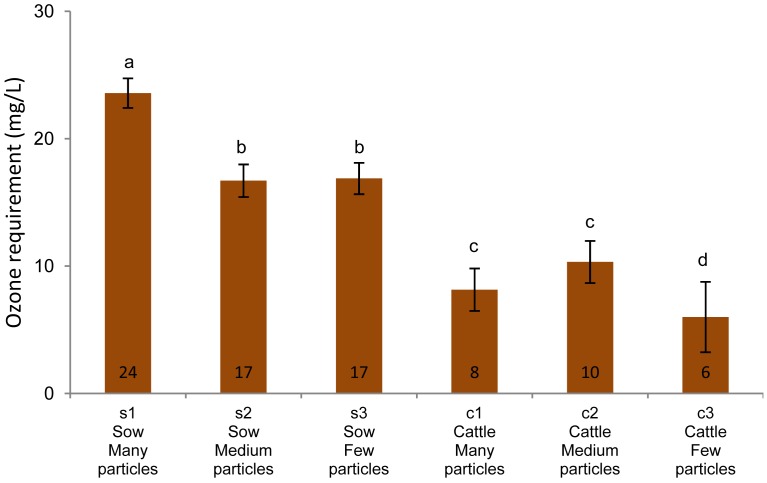
Ozone amount required to lower the gaseous H*_2_*S concentration above different manures to <0.05 ppm H*_2_*S. The error bars indicate standard deviations. Statistically equal results are indicated by the same letters above the columns.

**Figure 5.**
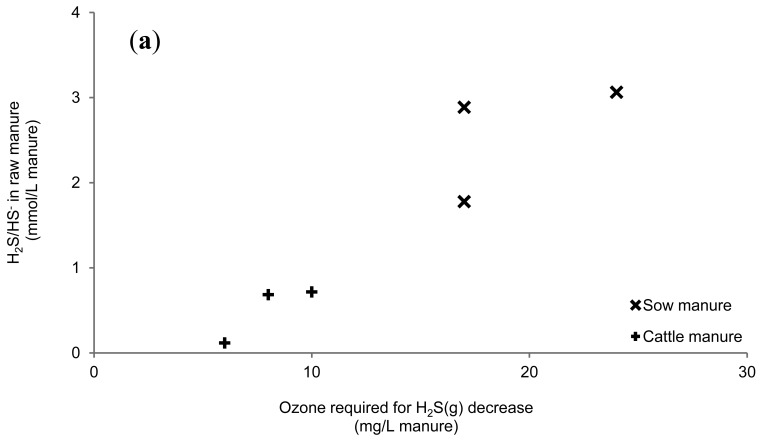

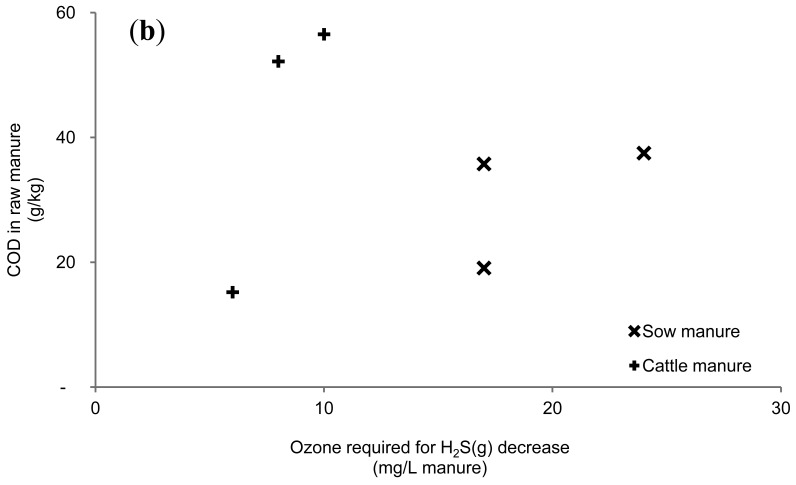
Relationship between ozone additions required to lower the gaseous H*_2_*S concentration (**a**) with total H*_2_*S/HS^−^ concentration in the raw manure, and (**b**) with the total chemical oxygen demand (COD) of the raw manure.

The total ozone requirement for minimizing the H*_2_*S emissions (Figure 4) increased as the total H*_2_*S/HS^−^ concentrations in the non-ozonated manure (Table 1) was increased (*R*^2^ = 0.89) (Figure 5(a)). Hence, ozone's initial reaction may be a reaction with reduced dissolved sulphur.

The very initial reactivity of ozone towards H*_2_*S and HS^−^ compared to the reactivity towards other dissolved species can be assessed based on existing kinetic data from the literature [13,22]. The time for reduction of the ozone concentration to 1/*e* (Eulers number) times the initial concentration, *i.e.*, the lifetime of ozone τ*_O3_*, with respect to dissolved species, can be estimated if the initial concentration of the dissolved species is known:

(1) $$\text{d}[\text{X}]/\text{dt}={\text{k}}^{2\text{nd}}[\text{X}][{\text{O}}_{3}]=\text{k}\prime [{\text{O}}_{3}];\text{k}\prime [{\text{O}}_{3}]={\text{k}}^{2\text{nd}}[\text{X}]$$

where k' is the pseudo-first-order rate constant, [X] denotes the concentration of the compound that reacts with ozone, and k^2nd^ denotes the second order rate constant for the reaction of ozone with compound X. Because the initial concentration of ozone is very low, the concentrations of other reactants in the manure can be assumed constant in a short initial time interval, and a pseudo-first-order expression for ozone's lifetime can be applied:

(2) $$\tau_{\text{o}3}=1/\text{k}\prime =1/({\text{k}}^{2\text{nd}}[\text{X}]$$

The calculated lifetimes are only approximations of the very initial reactivity, *i.e.*, until the reactions leads to reductions in concentrations of the species reactive towards O*_3_*. Despite this, it is however possible to assess the relative importance of various compounds for consumption of ozone.

The calculated initial ozone lifetime with respect to manure relevant species are the lowest for HS^−^ (Table 2). This supports the observed increase in the total ozone requirement at increasing H*_2_*S/HS^−^ concentration in the non-ozonated manures (Figure 5(a)). Consequently, it is assessed that only when the concentration of H*_2_*S/HS^−^ is lowered several orders of magnitude will ozone be available for degradation of other compounds. Non-dissociated phenols are, despite the low concentration, the largest competitor with HS^−^ for reaction with ozone, which in particular would be the case if the pH was higher. The calculated ozone lifetimes, however, indicate that it is very likely, that the added ozone oxidizes HS^−^ in the manure.

The calculated lifetime of ozone upon reaction with carboxylic acids, 4-methylphenol, other phenols and anions of phenols are high compared to the lifetime upon reaction with HS^–^ (Table 2). Hence, the main ozone consumption is not indicated to be caused by dissolved organic compounds. No relationship between ozone requirement and dry matter content or total chemical oxygen demand (COD) was observed (Figure 5(a), Table 1, Figure 4). Neither were dry matter and total COD entirely responsible for the ozone requirements within the manure types. This is in agreement with previous studies, in which only weak correlations were found between dry matter content and the oxidation level [23,24] and corroborates that ozone predominantly is consumed by H*_2_*S/HS^−^ as long as it is present.

If ozonation was the only reaction responsible for the H*_2_*S removal and if competing ozone reactions are assumed to be insignificant, the initial amount of the reduced sulphide (Table 1) should approximately be equal to the amount of ozone required to minimize H*_2_*S emissions (Figure 4). The molar ratio of ozone required for H*_2_*S elimination relative to the initial amount of total reduced sulphide were calculated to be in the range of 0.1 to 1.1 for the six batches of manures. Assuming a 1:1 stoichiometry of the reaction and no secondary reactions of importance, only in one batch (c3; molar ratio = 1.1) is the amount of ozone added sufficient to explain the removal of the reduced sulphide. Hence, the reduced sulphide is also removed by other mechanisms than ozonation.

Chemical or microbial oxidation by co-added oxygen (the ozone-enriched oxygen stream contained 1.5–2.2% O*_3_* and 97.8–98.5% O*_2_*) may cause the additional decrease in the H*_2_*S emissions [25–28]. Based on a previous study [28] it is estimated that <3% of the added O*_2_* is consumed by the slurry. Oxygen oxidation is observed in the control experiment in this study upon addition of pure oxygen (Figure 3). However, reaching the low H*_2_*S emission level required 0.2–0.7 L gas per L manure upon addition of the O*_3_* and O*_2_* gas, while it required approximately 1.4 L gas per L manure upon addition of pure O*_2_*. That is the approximately 2% O*_3_* in the added gas causes the reaction rate per gas volume to increase 2–7 times. Hence, chemical or biological oxidation by O*_2_* also contributes to reducing H*_2_*S concentration, but the reaction rate with O*_2_* is much lower than O*_3_*.

The total experimental treatment time was 10 to 160 min. The significant decrease of 99.5% in gaseous H*_2_*S concentration, however, occurred within 3–6 min. The short experimental duration and the peak in the removal rate are atypical for microbial oxidations. Hence, microbial oxidation is not likely to have caused the decrease in gaseous H*_2_*S concentration. Hence, the observed oxidation by O*_2_* were due to chemical oxidation.

**Figure 6.**
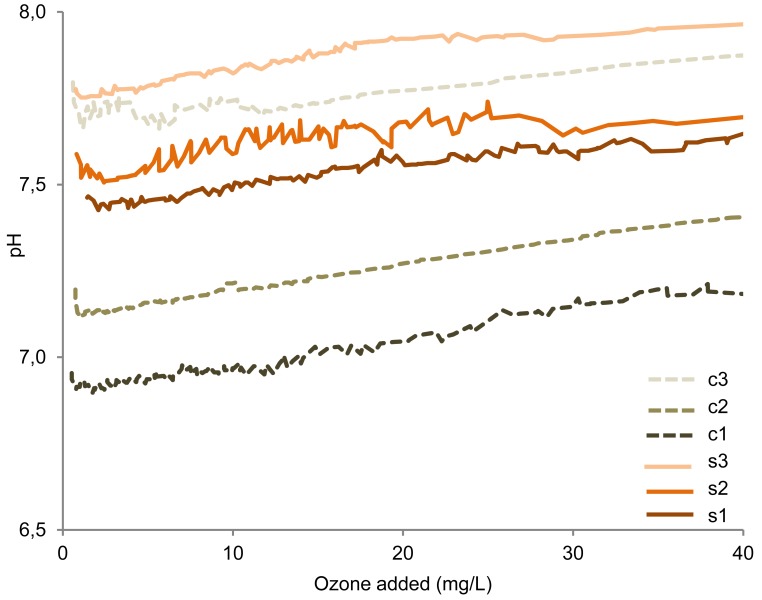
The pH increase as a function of ozone addition. Average trendline of the replicates for the individual manures.

An increase in pH and hence a decrease of dissolved H*_2_*S relative to HS^–^ would also lead to lower gaseous emissions of H*_2_*S. A continuous pH increase, due to the emission of e.g., H*_2_*S and CO*_2_*, was in fact observed during addition of the ozone enriched oxygen (Figure 6), with the average pH increase being 1 pH unit per 230 mg O*_3_* added per L manure. However, the pH increase observed simultaneously with the decrease in the H*_2_*S emissions (–200 mV to −80 mV) was only 0.01–0.05 pH units. Based on calculations of the H*_2_*S/HS^–^/S^2–^ distribution from pK*_a_*-values and observed pH, it is estimated that the pH increase could only result in a reduction of the emissions by ∼0–7%. Hence, the pH increase cannot account for the observed decrease of the H*_2_*S emissions.

Volatilization of the H*_2_*S when adding gas (flushing) could cause the additional decrease in the gaseous concentration of H*_2_*S above the manure. This is observed not to be the case, as the control treatment with N*_2_* addition did not change the H*_2_*S emission (Figure 3). This can also be estimated from the volume of added O*_2_* gas, the Henry's law constant, pK*_a_* of H*_2_*S and pH. In all cases, the relative amount of total reduced sulphide removed by gas flushing is estimated to be <5%. Hence, the flushing cannot account for additional removal of H*_2_*S.

In conclusion, it is ozone and oxygen as oxidizing agents that causes the minimization of H*_2_*S emissions. This is well aligned with the observed correlation between increase in redox potential and decrease in H*_2_*S emissions.

### 3.4. Assessment of Required Dose of Oxidizing Agent

It has previously been difficult to accurately calculate the exact amount of oxidizing agent required to minimize H*_2_*S emissions from manures [1,9,14,15]. The present study indicates some useful methods for determining the optimal amount of oxidation agent needed.

The amount of ozone required for H*_2_*S removal was observed to correlate with the H*_2_*S/HS^−^ content of the non-ozonated manure. Thus, as an alternative to monitoring the actual H*_2_*S emissions continuously, the H*_2_*S/HS^−^ concentration of the raw slurry may be analyzed.

The decrease in H*_2_*S emissions was observed to occur simultaneously with a rise in redox potential from ‐200 mV to ‐80 mV (Figures 2 and 3). Hence, in batch oxidation operations, it should be possible to apply the redox potential at termination of the rapidly changing redox phase and initiation of the stable redox phase (approximately −10 mV) in order to adjust the dose of oxidizing agent. This can be calculated as the intercept of the linear regression for the two redox potential phases (Figure 1).

For the six manure batches in this study, the required amount of ozone is estimated to be 6–24 mg O*_3_*/L manure (Figure 4). In previous studies of manure, amounts of 250–3,000 mg O*_3_*/L have been added to eliminate emissions of odorous compounds by ozonation [1,8,11,12]. These studies focused on compounds other than H*_2_*S, but the present study demonstrates that if the main objective of oxidation is to eliminate H*_2_*S emissions instantaneously, much lower doses can be used when applied with proper process control.

Monitoring redox continuously to assess the appropriate dose of oxidizing agent has been demonstrated to be applicable to two manures in three particle size ranges (Table 1), which covered different manure characteristics: both cattle and pig manure, and both high and low dry matter contents, total COD contents, redox potentials, H_2_S/HS^−^ concentrations, and H_2_S− to-HS^−^ ratios, and different particle size ranges. Hence, the control method is robust with respect to different manure characteristics. It is therefore applicable independently of farm operation and the continuously varying manure characteristics. This indicates that similar relations may be expected in other media than manure, such as drinking water and wastewater [16,29]. However, it is possible that these media contains oxidizable compounds competing with H_2_S/HS^−^ in terms of O_3_ and O_2_ reactivity, resulting in less distinct changes in redox. Analogous relations between redox potential and reduction in H_2_S emissions may also be expected with respect to oxidation using other oxidizing agents than ozone and oxygen, such as aeration, hydrogen peroxide treatment, permanganate treatment, Fenton's process, photo-oxidation, and anodic oxidation [2,10,24,30–32].

## 4. Conclusions

The oxidation curves (amount of oxidizing agent *vs.* redox potential) of the tested animal manures exhibited similar rises in redox potential upon addition of ozone enriched oxygen. A rapid increase in redox potential was observed at −200 mV to −80 mV. Simultaneously, the concentration of H*_2_*S above the manure decreased by 99.5%. The H*_2_*S removal was caused by ozone and oxygen oxidation.

Continuous redox potential measurement, *i.e.*, termination of the rapid redox potential rise, has proved applicable for process control upon oxidation of manure. The application is robust with respect to different manure characteristics. It may also by applicable for other combinations of media and oxidizing agents. Hence, applying continuous monitoring of redox potential for process control of oxidation treatment is predicted to enable more cost-effective treatments and increased guarantee of malodourous H*_2_*S emissions abatement.

## Figures and Tables

**Table 1.** Characteristics of untreated animal slurry batches. Standard deviations (*n* ≥ 3) are indicated in brackets.

**Manure ID**	**Animal origin**	**pH**	**Particle sizes [mm]**	**Dry matter [g/kg]**	**COD [g/kg]**	**H***_2_***S/HS**^−^ **[mM]** ^1^
s1	Sow	7.4	<5	58 (0)	38 (1)	3.1 (0.1)
s2	Sow	7.5	<0.3	30 (0)	36 (1)	2.9 (0.1)
s3	Sow	7.7	<0.2	19 (0)	19 (1)	1.8 (0.1)
c1	Dairy cattle	6.9	<5	74 (0)	52 (7)	0.69 (0.03)
c2	Dairy cattle	7.1	<0.06	49 (0)	57 (1)	0.72 (0.01)
c3	Dairy cattle	7.7	<0.035	16 (0)	15 (0)	0.12 (0.01)

1 S^2−^ are assumed absent, because pKa(HS^−^/S^2^)^−^ is 13.8, and because the rate constant(HS^−^/S^2−^) is ∼10 times lower than expected for a diffusion controlled reaction.

**Table 2.** Estimated lifetime of ozone upon reaction with manure components present in high concentration and with large reaction rate constants.

**Compound**	**Rate constant** ^1^ **[M**^–1^**s**^–1^**]**	**pKa**^2^	**Concentration**^3^ **[mM]**	**τ***_O3_*^4^ **[s]**
H*_2_*S	3 × 10^4^	7.00	0.02–0.91	0.4–1.6 × 10^–1^
HS^−^	3 × 10^9^	7.00	0.1–2.2	0.2–3.4 × 10^–6^
NH*_3_*	2 × 10^1^	9.25	0.7–5.3	0.9–6.9 × 10^1^
4-methylphenol	3 × 10^4^	10.2	1	3 × 10^–2^
4-methylphenolate	∼1 × 10^9^	10.2	0.0005	2.3 × 10^–4^
Butanoate	<4 × 10^–2^	4.8	1.5–9.8	0.3–1.6 × 10^4^

1 Source: [13,22]. For 4-methylphenolate, the value for phenolate has been used;

2 pKa-values for corresponding acids are shown;

3 Concentration [X] range based on measured sum of acid and base concentration, the pKa value and the measured pH (Table 1) in the six batches. The presented 4-methylphenol and 4-methylphenolate concentrations were the maximum in [21];

4 Ozone's lifetime upon reaction with the compound is calculated with Equation (1).
